# An improved method for genome wide DNA methylation profiling correlated to transcription and genomic instability in two breast cancer cell lines

**DOI:** 10.1186/1471-2164-10-223

**Published:** 2009-05-13

**Authors:** Jian Li, Fei Gao, Ning Li, Shengting Li, Guangliang Yin, Geng Tian, Shangang Jia, Kai Wang, Xiuqing Zhang, Huanming Yang, Anders Lade Nielsen, Lars Bolund

**Affiliations:** 1 Institute of Human Genetics, University of Aarhus, The Bartholin building, DK-8000, Aarhus C, Denmark; 2 Beijing Institute of Genomics, Chinese Academy of Sciences, Beijing, 101300, PR China; 3 Beijing Genomics Institute, Shenzhen, Guangdong, 518083, PR China; 4 Graduate School of Chinese Academy of Sciences, Yuquan Road 19A, Beijing, 100039, PR China; 5 Bioinformatics Research Center (BiRC), University of Aarhus, Hoegh-Guldergs Gade 10, Building 1090, DK-8000, Aarhus, Denmark; 6 Beijing Genomics Institute, B6 Industrial Zone, Beijing, 101300, PR China; 7 Danish Centre for Translational Breast cancer research (DCTB), Strandboulevarden 49, DK-2100, Copenhagen, Denmark

## Abstract

**Background:**

DNA methylation is a widely studied epigenetic mechanism known to correlate with gene repression and genomic stability. Development of sensitive methods for global detection of DNA methylation events is of particular importance.

**Results:**

We here describe a technique, called modified methylation-specific digital karyotyping (MMSDK) based on methylation-specific digital karyotyping (MSDK) with a novel sequencing approach. Briefly, after a tandem digestion of genomic DNA with a methylation-sensitive mapping enzyme and a fragmenting enzyme, short sequence tags are obtained. These tags are amplified, followed by direct, massively parallel sequencing (Solexa 1G Genome Analyzer). This method allows high-throughput and low-cost genome-wide DNA methylation mapping. We applied this method to investigate global DNA methylation profiles for widely used breast cancer cell lines, MCF-7 and MDA-MB-231, which are representatives for luminal-like and mesenchymal-like cancer types, respectively. By comparison, a highly similar overall DNA methylation pattern was revealed for the two cell lines. However a cohort of individual genomic loci with significantly different DNA methylation status between two cell lines was identified. Furthermore, we revealed a genome-wide significant correlation between gene expression and the methylation status of gene promoters with CpG islands (CGIs) in the two cancer cell lines, and a correlation of gene expression and the methylation status of promoters without CGIs in MCF-7 cells.

**Conclusion:**

The MMSDK method will be a valuable tool to increase the current knowledge of genome wide DNA methylation profiles.

## Background

One key component of the cancer epigenome is an altered DNA methylation pattern by global hypomethylation and promoter localized hypermethylation [1]. These methylation changes can result in an alteration in structure and function of DNA, such as unwanted activation of repeat elements, abnormal transcriptional regulation of genes involved in cancer initiation and progression, and predisposition to genomic instability through disruption of chromosome replication control [2,3].

Numerous studies have paid attention to the difference of DNA methylation profiles between malignant breast cells and normal control, regardless of their origins [4,5]. Molecular subtype taxonomy of breast cancers and breast cancer cell lines is more and more important, and the identified new biomarkers that inflect cell origin might be promising targets for disease treatment [6-8]. MCF-7 and MDA-MB-231 are widely used breast cancer cell lines in cancer research [9]. Both cell lines were established from metastatic cells in pleural effusions collected from two individual ductal invasive breast cancers [9,10]. MCF-7 is classified as a luminal (epithelium)-like cell line with relatively low invasive potential, whereas MDA-MB-231 is a mesenchymal-like cell line which is highly invasive [6,10]. Comparison of the two cell lines in terms of DNA copy number variation (CNV) and gene expression profiles have been performed [6,11]. However, a comprehensive picture of DNA methylation patterns, DNA copy number alterations and gene expression levels genome wide for the two cell lines remains to be established.

A detailed exploration of the role of DNA methylation in tumorigenesis depends on sensitive methods to precisely describe DNA methylation states genome wide. Methylation-specific digital karyotyping (MSDK) is one such powerful method [12,13], which combines the use of methylation-sensitive restriction enzyme digestion and sequencing by a SAGE-like method to achieve genome-wide DNA methylation maps [12,13]. However, this technique is limited by a relatively low throughput and a high cost of sequencing. Solexa 1G Genome Analyzer is a new generation sequencer which can perform massively parallel signature sequencing. Thus, it has been applied in epigenetic studies to improve currently existing techniques [14-16]. In the present study, we establish a method, modified methylation-specific digital karyotyping (MMSDK) on the basis of the combination of original MSDK [12,13] and the new sequencing technique using Solexa 1G Genome Analyzer. In the original MSDK method ditags are produced followed by clone sequencing. In MMSDK tags are directly amplified by PCR using a pair of universal primers and subsequently sequenced using a Solexa sequencer. Finally, tags (reads) are mapped back to the human genome. We chose MCF-7 and MDA-MB-231 as representatives for luminal-like and mesenchymal-like subtypes, respectively, and used these cell lines for comparative analysis by MMSDK, array comparative genomic hybridization (aCGH) and gene expression microarray analysis to explore the correlation between DNA methylation, genomic stability and gene expression. We demonstrate that MMSDK is a genome-wide, high-throughput and cost-effective method to analyze DNA methylation in large genomes such as the human and that MMSDK can be used to reveal connections between genomic, epigenetic and transcriptional features.

## Methods

### Biological material

The two breast cancer cell lines MCF-7 and MDA-MB-231 were obtained from the European Collection of Cell Culture. Cells were routinely cultured in DMEM medium supplemented with 10% fetal bovine serum and collected during the growth phase before confluence.

### MMSDK

**Figure 1:**
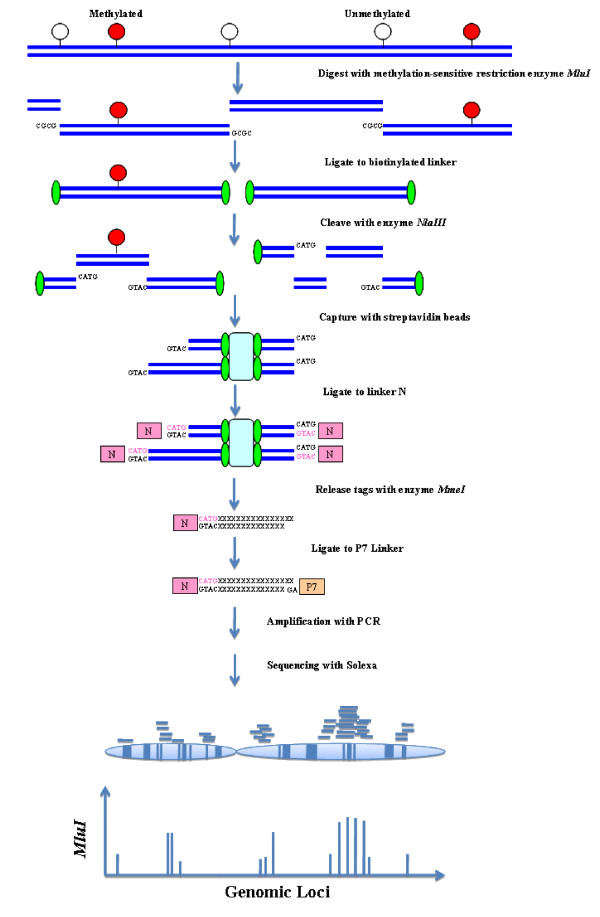
**The strategy of MMSDK**. Schematic presentation of the MMSDK method. Details of the method are described in the text.

MSDK [12,13] is a modification of the original digital karyotyping technique [17]. In this study, we developed MMSDK as a combination of the original MSDK and Solexa sequencing (Fig 1). The procedures prior to tag concatenation were similar to those described originally for MSDK [12,13]. Briefly, DNA was isolated from the cell lines using DNeasy^® ^Blood & Tissue Kit (QIAGEN) following manufacturer's protocol. Genomic DNA was digested with methylation-sensitive mapping enzyme *MluI *(New England Biolabs). *MluI *has two CpG sites in its recognition sequence, ACGCGT, and therefore its recognition site is preferentially located in CpG islands (CGIs). Digested DNA was ligated to biotinylated linkers and fragmented by *NlaIII *(New England BioLabs) cleavage. Because *MluI *only cuts unmethylated regions, binding of DNA fragments to streptavidin-conjugated magnetic beads will separate the unmethylated and methylated fragments. Because of a potential risk that digested DNA without biotinylated linkers may be unspecifically bound by streptavidin-conjugated beads, we performed a parallel control experiment instead using DNA fragments without biotinylated linkers. Bound DNA was ligated to another linker (N) containing a *MmeI *restriction enzyme recognition site, and then digested with *MmeI *(New England Biolabs) that generates short sequence tags (16–17 bp, due to enzyme cut floating). Instead of the concatenation of tags and clone sequencing, tags were ligated with P7 linker and amplified by PCR with primers N and P7. To avoid the bias from PCR, amplification was stopped after 15 cycles. The sequences of the linkers and primers are available in Additional file 1.

### Clone sequencing

Conventional clone sequencing was carried out prior to Solexa sequencing. Briefly, PCR products from the two cell lines and the control were ligated with T-easy vector (Promega), and transformed into competent DH5α bacteria (Takara). Plasmid DNA was purified from 19 clones of each PCR product (MCF-7, MDA-MB-231 and the control) and analyzed by conventional sequencing.

### Sequencing using Solexa 1G Genome Analyzer

Sequencing-By-Synthesis (SBS) was performed for the generation of tags using Illumina Cluster Generation (Illumina) and 1G Genome Analyzer (Illumina) according to the manufacturer's instructions [18]. All reagents for the sequencing process were purchased from Illumina Inc. Briefly, the PCR product was ligated with adaptors (Sequences of adaptors and PCR primers are available in Additional file 1). DNA fragments were amplified by PCR using Phusion polymerase (Finnzymes). PCR products were purified using a QIAquick MiniElute Kit (Qiagen). We performed cluster generation and standard cycle of sequencing on the Illumina cluster station and 1G analyzer following the manufacturer's instructions. Finally, the tag sequences were stored as libraries for each cell line.

### Tag mapping and statistical analysis

We extracted 16–17 bp tags from all reads obtained by sequencing. Because of the risk that the length of 16–17 bp is too short to obtain high confidence whole genome wide mapping, we developed a mapping process based on the simulation of enzyme cutting of the DNA. The human genome sequence and mapping information (Santa Cruz human genome assembly (hg18), March 2006) was downloaded from the University of California, Santa Cruz Genome Bioinformatics Site [19] and a virtual *MluI *and *NlaIII *cutting DNA fragment library was constructed as follows: we located the predicted *MluI *sites, identified the nearest *NlaIII *sites in both directions and sampled the derived DNA fragments as the reference for mapping. MAQ (Mapping and Assembly with Qualities) was used to map all tags back to this library [20,21]. We defined high-confidence mapped tags as those with a mapping quality score more than 20. In order to identify genes neighboring the *MluI *sites (to correlate their expression with the degree of methylation), we removed all virtual tags with low confidence mapping results (mapping quality score < 20) in the genome to ensure unambiguous mapping of the data. As the description in MAQ manual, a tag with mapping quality 20 should have 1% error rate in principle and a tag with quality higher than 20 should have an alignment error rate below 0.1% on average. SNPs and sequencing errors are considered in calculating this score. For the analysis of the repeat families and genome wide overview scanning, we considered all tags mapped back to the genome with a tolerance of low-confidence mapping. We used all mapped tags for determination of their chromosome location, then normalized tag numbers to address the potential problem of uneven total tag numbers between the libraries. The normalized libraries were used for comparison of the two cell lines and calculation of *P*-values. To analyze the significance of the differences in DNA methylation between two cell lines, Z-score test was used to calculate the *P*-value for each *MluI *site in the genome by means of pair-wise comparison. False discovery rate (FDR) control was added for the correction of the *P*-values to address the multiple comparisons problem of the high-throughput techniques. FDR controls the expected proportion of incorrectly rejected null hypotheses (type I error). It is a less conservative procedure for comparison, with greater power than familywise error rate (FWER) control, at a cost of increasing the likelihood of type I error. The corresponding gene annotations are from the National Center for Biotechnology Information "The Reference Sequence (RefSeq)" database [22].

### qPCR

A qPCR assay was designed for the validation of the methylation results from MMSDK (see Additional file 2). Briefly, genomic DNA from the two cell lines was equally divided into two parts. One portion was digested with *MluI *and the other portion served as an undigested control. The digested and undigested DNA samples were amplified by qPCR using locus-specific primers that flank a given *MluI *restriction site in the human genome. The methylation state of a given genomic locus is determined in a manner that depends on restriction digestion of unmethylated sequences using *MluI *and the resulting failure to PCR amplify the digested fragements. Only the fragements containing methylated *MluI *site are protected from the digestion and, thus, serve as template for amplification. All PCR primers were designed to generate fragments of about 100 bp. A genomic region containing a *HindIII *cutting site (AAGCTT) but no *MscI *cutting site (TGGCCA) was selected as control. An equal amount of this template was digested by *HindIII *or *MscI*. Through quantitive comparison between the two sets of PCR products, the methylation state of the *MluI *site for a given genomic locus will be identified. The selected genomic loci for qPCR validation and their corresponding primer sequences are available online (see Additional file 3).

### Bisulfite treated PCR and clone sequencing

We applied MethyPrimer website service to design bisulfite treated PCR primers. Genomic DNA in both breast cancer cells was conversed using EpiTect Bisulfite Kit (Qiagen), followed by PCR amplification, catalyzed by Taq clone polymerase (Invitrogen). The PCR program is as follows: denature at 95°C for 4 min, followed by 5 cycles: denature at 95°C for 30 sec, anneal at 56°C (variable according to primers' Tm) for 90 sec, and extend for 2 min, followed by 25 cycles: denature at 95°C for 30 sec, anneal at 56°C (variable according Tm for primers) for 90 sec, and extend for 90 sec. PCR products were purified using GFX PCR DNA and Gel Band Purification Kit (GE Healthcare) and performed transformation using TOPO TA Cloning for Sequencing Kit (Invitrogen). Single colonial bacteria were picked up from LB plate for culturing. Plasmid DNA was purified using GenElute Plasmid Miniprep Kit (Sigma) from bacteria cluture. Plasmid DNA for each genomic locus was sequenced using dideoxy chain termination method with ABI 3730XL 96-capillary sequencer. Sequencing was performed by MWG in Ebersberg. The outcome of bisulfite clone sequencing result for each genomic locus was visualized by CpG viewer [23]. The information of the genomic loci and the primers is available in Additional file 3.

### Array CGH

Array CGH was performed as described previously [24]. Briefly, CytoChip v2.0 (BlueGnome) with BAC clone human DNA fragments, representing the whole genome with 0.5-Mb resolution on average, was used to detect copy number variation (CNV) for both cell lines. A dye-swap method was applied in the array CGH. Cell line sample and reference (pooled healthy females) DNA was labeled with Cy3-dCTP and Cy5-dCTP (Amersham Biosciences), respectively, using a random priming method with the labeling kit BioPrime^® ^DNA labeling system (Invitrogen), and the reverse labeling for sample and reference DNA was performed as well. The mixture of the differentially labeled cell line DNA (22~24 ug) and reference DNA (22~24 ug), together with 230 ul (1 mg/ml) Cot-1 DNA (Invitrogen) for blocking hybridization to repetitive sequences, was ethanol precipitated and resuspended in hybridization mix (50% formamide, 10% dextran sulfate, 2XSSC, 1%–4% (v/v) SDS, heering sperm DNA (10 mg/ml) (Invitrogen), and salmon sperm DNA (10 mg/ml) (GIBCOBRL, Life Technologies)) to a total volume 130 ul. After denaturing at 75°C for 15 min, the DNA mixture was incubated at 37°C for 1.5 hours to allow regression annealing of repetitive sequences. The pre-hybridization, hybridization and washing were performed using HS 4800 Pro hybridization station (TECAN). After washing with PBS, the hybridization mix was added to the array. Tumor and reference DNA co-hybridize the denatured target DNA on the CytoChip slide with relative kinetics dependent on CNV. After hybridization, the slide was washed with a series of solutions: PBS and 0.05% Tween20 at 37°C for 3 min; 0.1 × SSC at 54°C for 3 min; PBS and 0.05% Tween20 at 23°C for 80 sec; 0.2 × SSC for 30 sec. Finally, the slide was dried by blowing nitrogen. The array was imaged in a GenePix 4000 B laser scanner (Axon Instruments). After optimizing exposure time, the array was scanned in Cy3 and Cy5 channels, respectively. Two single-channel 16-bit images were combined for analysis using the image-analysis software "BlueFuse" v3.5 (BlueGenome). The thresholds for deletion and amplification were -0.299 and 0.299, respectively, by default. The breakpoints in the genome were determined by the flanking clones that located the boundaries of the aberration regions. The BAC clone positions information is based on NCBI Build 36.

### Gene expression profiling

Total RNA was extracted from frozen cell lines with TRI Reagent (Sigma) according to the manufacturer's protocol. The integrity of the extracted RNA extract was checked by agarose gel electrophoresis, and the concentration of RNA was estimated by spectrophotometry. Gene expression profiles were obtained by array hybridization using Human U133 Plus 2.0 (Affymetrix), which can estimate the expression level of 47,400 transcripts and variants. cRNA preparations, hybridizations, washing and detection were performed at Aros Biotechnology, Aarhus, Denmark, according to the manual of Affymetrix.

## Results

### MMSDK

To elucidate genome wide DNA methylation patterns we developed a modified version of MSDK, MMSDK. To distinguish methylated from unmethylated cytosines we selected the *Mlu1 *restriction enzyme which is a methylation sensitive six-bp cutter. For further DNA fragmentation and establishment of a second anchor for linker ligation, DNA also was digested with *NlaIII *which is a methylation insensitive restriction enzyme. *In silico *analyses showed that there are 21309 recognition sites for the *MluI *restriction enzyme in the human genome. We performed a digital digestion of the human genome with *MluI *and *NlaIII *under the hypothesis that all cytosines are unmethylated. We notice the presence of 369 *MluI *fragments lacking internal *NlaIII *recognition sites. The distribution of the lengths of the theoretically generated *MluI/NlaIII *fragments is presented in Additional file 4. The majority of fragments are shorter than 1000 bp, with a frequency peak at 50–150 bp. There are 3154 *MluI *recognition sites in CGIs defined by three criteria (GC content > 50%, ratio of the observed CpGs to the expected CpGs > 0.6, length > 400 bp), accounting for 14.8% of all *MluI *recognition sites in the human genome. Furthermore, our approach also enables us to determine the methylation state of CpGs in repeat sequences. According to Repeatmasker [25], 353 (1.66%) of the *MluI *sites are located within repeat sequences in the human genome.

The detailed protocol for the MMSDK procedure is described in the Methods section. Prior to large-scale Solexa sequencing, we performed clone sequencing to check the outcome of MMSDK. A total of 57 clones (19 each from MCF-7, MDA-MB-231, and the control) were selected for sequencing. 18 clones from MCF-7 and 19 clones from MDA-MB-231 presented unique human genomic sequences with the expected CATG motif in the end. All sequences from the 19 clones from the control solely contained linker sequence or less than 12 bp inserted sequence without CATG motif. By the Solexa sequencing in MMSDK, we obtained 5432906 and 5636928 sequenced tags with 16–17 bp length from MCF-7 and MDA-MB-231 cells, respectively (see Additional file 4). More than 94% of the tags obtained from both cell lines could be mapped back to *MluI *recognition sites in the human genome. All information of the obtained MMSDK libraries for the two examined cell lines is available in Additional file 5. Our method allows us to reveal significantly different methylation of individual genomic loci. By comparison of methylation libraries from the two cell lines, we identified the individual genomic loci that have significantly different DNA methylation status (FDR *P*-value < 0.05) in the two cell line libraries (see Additional file 5).

### Overview of the DNA methylation profiles in the two cancer cell lines

At first, we compared the methylation profiles of promoters, introns, exons and CGIs (not located to known promoter sequences) for both cancer cell lines using a T-test statistical method (Table 1). The two cell lines showed generally similar DNA methylation states in the examined genomic category of sites. This is in agreement with analysis by Shann et al. examining DNA methylation in MDA-MB-231 and MCF-7 cells by a different technical approach [4]. In an alternative analysis, we collected the genomic loci with significantly different methylation states (FDR<0.01) between the two cell lines. These genomic sites were then divided into different groups according to their genomic category (Table 2). Finally, we compared the methylation states for the different genome categories between the two cell lines using T-test (Table 2). Except for the group of genomic sites representing introns, the rest of the genomic categories had similar DNA methylation profiles in the two cell lines.

**Table 1:** Comparison of the methylation states in various genomic categories

			High confidence mapped tags			All mapped tags		
			
		Site counts^1^	P-value^2^	Mean^4 ^of MCF-7	Mean of MDA-MB-231	P-value^3^	Mean of MCF-7	Mean of MDA-MB-231
	No CGI	596	0.8928	200.1946	194.615	0.7313	412.307	386.2217
	
Promoter	CGI	1042	0.2108	501.2428	403.7937	0.09058	738.3695	593.3216

Intron		8213	0.9325	111.504	107.5048	0.8821	236.1427	228.3417

Exon		937	0.6431	63.10886	56.21106	0.7668	200.3436	186.15013

CGI(No promoter)		2113	0.9173	158.5939	156.5405	0.4425	325.3417	304.564

Table 1 presents a comparison of the methylation states of promoters (including the promoters with CGIs and without CGIs), introns, exons and CGIs (no promoters) for both cell lines using T-test statistical method, which provides an overview of the difference of the methylation state between two cell lines. 1 The information of the number of sites in different genomic categories is presented. 2–3 The statistical results of the difference of methylation states in different genomic categories between two cell lines are presented, considering high confidence mapped tags and all mapped tags, respectively. 4 The mean of the number of tags for the corresponding cell line in a given genomic category.

**Table 2:** Comparison of the methylation states based on significantly different individual genomic sites

			High confidence mapped tags			All mapped tags		
			
		Site counts^5^	*P*-value	Mean of MCF-7	Mean of MDA-MB-231	*P*-value	Mean of MCF-7	Mean of MDA-MB-231
	No CGI	12	0.8585	524.3333	617.5263	0.9704	791.1667	768.7776
	
Promoter	CGI	17	0.2384	1578.882	550.6183	0.2505	2038.7647	850.4476

Intron		52	0.008962	350.1538	1286.1792	0.01392	562.4423	1503.848

Exon		15	0.378	854.0667	396.7598	0.3564	1090.0667	518.7343

CGI(No promoter)		27	0.3531	470.037	896.2317	0.4785	912.926	1292.6098

The individual genomic sites with significantly different number of tags between two cell lines were collected and classified to the corresponding genomic categories. Then a T-test statistical analysis was performed to test the difference of the methylation states of promoters (including the promoters with CGIs and without CGIs), introns, exons and CGIs (no promoters) between both cell lines. 5. The number of individually significantly different genomic sites involved for a give category is shown. The meanings of the *P*-value and mean in Table 2 are the same as in Table 1.

**Figure 2:**
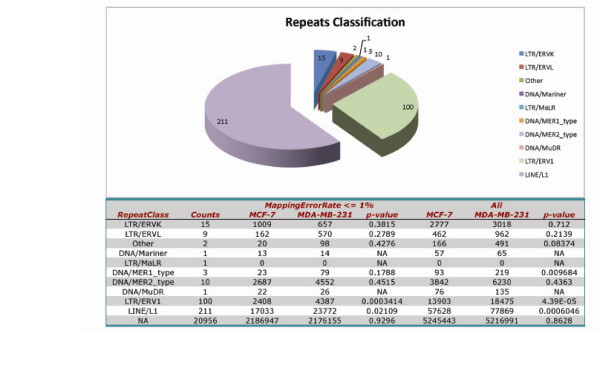
**Distribution of *MluI *sites in repeat sequences and summary of sequence tag information**. The distribution of *MluI *recognition sites in different classes of repeat sequences is presented in the top pie chart, and the bottom table summarizes the counting result of the tags collected for different classes of repeat sequences of the MCF-7 and MDA-MB-231 cell lines.

Our MMSDK method also allows investigation of the DNA methylation status for repeat sequences. As described above, a T-test was performed for the comparison of the distribution of unmethylated sites in different repeat sequence categories in the two cancer cell lines. The summary of the collected tags information in repeat sequences for the cell lines is presented in Fig 2. Notably, a significant difference between two cell lines in the methylation level of long interspersed nuclear elements (LINE/L1) was observed (Fig 2).

### Validation of MMSDK results with qPCR

In order to validate the results of MMSDK by an alternative method, we randomly selected a cohort of 12 genomic loci to test their DNA methylation states by a qPCR based method (see Methods section). The information on the selected genomic loci is provided in Additional file 3. Of the 7 loci with a relatively high number of tags in MCF-7 cells the qPCR data were consistent with the MMSDK data for 5 loci (see Additional file 6). Of the 5 loci with a relatively low number of tags in MCF-7 cells the qPCR data were consistent with the MMSDK data for 4 (see Additional file 6). For MDA-MB-231 genomic DNA analysis, a general correlation between MMSDK data and the qPCR analysis was also present albeit with some more inconsistencies between the two methods. Thus, qPCR analysis largely confirmed the MMSDK results, but we notice that genomic amplifications, efficiency of enzyme digestion, and bias from PCR can result in differences between the two methods.

### Validation of MMSDK results with bisulfite treated PCR and clone sequencing

The methylation statuses of the five *MluI *genomic loci were confirmed by bisulfite treated PCR and clone sequencing. These five randomly selected genomic loci are representative for the following situations in our study: high-level methylated sites for both cell lines (genomic loci 1, 2a and 2b); high-level unmethylated sites for both cell lines (genomic locus 3) and the sites with relatively higher methylated status in one cell line than the other (genomic locus 4). There are 6 and 7 clones presenting methylated for genomic locus 1 in MCF-7 and MDA-MB-231, respectively, (according to 50 tags and 12 tags for genomic locus 1 in MCF-7 and MDA-MB-231, respectively). Similarly, all 7 clones showed methylated in both cell lines in genomic loci 2a and 2b (according to 10 tags in both genomic loci 2a and 2b in both cell lines). All 7 clones presented unmethylated in both cell lines in genomic loci 3 (according to 7082 and 7546 tags in MCF-7 and MDA-MB-231, respectively). Six clones were unmethylated and 7 clones methylated in MCF-7 and MDA-MB-231, respectively (according to 4514 and 96 tags collected in MCF-7 and MDA-MB-231, respectively). Thus, bisulfite treated PCR and clone sequencing result also largely confirmed the reliability of MMSDK. The result of bisulfite treated genomic sequencing is presented in Additional file 7.

### Array CGH

**Figure 3:**
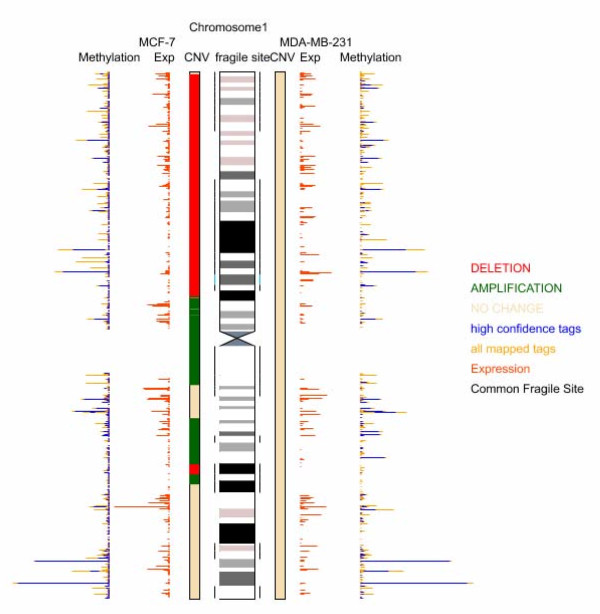
**The methylation profile, gene expression profile and DNA copy number variation of loci on Chromosome 1**. Chromosome 1 was selected as a representative of the chromosome set. Similar information for the other chromosomes is presented in Additional file 8. A diagram of chromosome 1 is located in the middle. The common fragile sites are symmetrically indicated with thin solid black lines (the rare fragile sites marked with blue). The corresponding data for MCF-7 and MDA-MB-231 are presented to the left and the right sides of the chromosome, respectively. The DNA copy number variation, gene expression and methylation status are shown consecutively. For DNA copy number variation, green, red and mild yellow mean amplification, deletion and no change, respectively. The length of the red bars that are vertical to the chromosome indicates the expression level for the corresponding genes. The length of bars located the most outside of the figure represents the methylation extent for the corresponding *MluI *sites on the chromosome. Blue indicates tags mapped with high confidence (mapping quality more than 20) and yellow represents all mapped tags.

The profiles of genomic CNV for the MCF-7 and MDA-MB-231 cell lines are shown in Fig 3 and in Additional file 8. The dye-swap method robustly confirmed aberrations. MCF-7 and MDA-MB-231 cells both have overt genomic instability. The main genomic aberrations in MCF-7 are deletions on 1p, 6q, 8p, 11q, 13q, 16q, 18q and chrX, and amplifications on 1q, 8q, 9p, 17q and 20q. In contrast, amplifications on 6p, 8q, 11q, 12q and 19p, and deletions on 2q, 3q, 6q, 7q, 8p, 12p, 13p 15p, 18q and chrX were presented in MDA-MB-231. The deletion and amplification regions accounted for 33.78% and 8.34% in the whole genome in MDA-MB-231, respectively. For MCF-7 cells, the percentages of the deletion and amplification regions in the genome were 19.58% and 14.58%, respectively. The breakpoints in the genome for two cell lines were revealed by means of identification of the color-change points (points flanked by two adjacent DNA fragments indicating different CNV) in Fig 3. Fragile sites (cytogenetically defined) are also indicated on the chromosomes (Fig 3). In our study, 20% and 22% of the genome breakpoints were located in the neighborhood of common fragile sites in MCF-7 and MDA-MB-231, respectively.

### Gene expression

The expression levels of 47,400 transcripts and variants were screened with Affymetrix microarray technology for both cell lines. There were 2345 transcripts having significantly increased expression in MCF-7 cells compared to MDA-MB-231 cells. 328 of these transcripts were also described in the study of Charafe-Jauffret et al. defining a gene expression profile in luminal-like breast cancer cellular subtypes [6]. The expression of 2157 transcripts was increased in MDA-MB-231 cells compared to MCF-7 cells. 387 of these transcripts were also described in the study of Charafe-Jauffret et al. to define a gene expression profile in mesenchymal-like cellular subtypes [6]. The gene expression data for the two cell lines and a description of the genes with significantly different expressions between the two cell lines are available in Fig 3 Additional file 5, Additional file 9 and Additional file 10. The microarray data have been deposited in gene expression omnibus GEO database [26]. The GEO accession number is GSE12199.

### Combinatory analysis of DNA methylation state, genomic stability and gene transcription

We performed genome-wide joint analysis of DNA methylation, genomic stability and gene transcription based on the three levels of global features revealed by MMSDK, aCGH and gene expression microarray analysis, respectively. We counted the number of tags from MMSDK and mapped them to the human genome including all predicted CpG islands (CGIs), considering *MluI *and *NlaIII *recognition site locations. Taking chromosome 1 as an example (Fig 3), the information of the numbers of tags, CNV status, and the gene expression level is displayed in the corresponding genomic loci on the displayed chromosome. The length of the bars labeled with blue and dark yellow in Fig 3 represents the quantitation of high-confidence mapped tags and all mapped tags, respectively. The length of the bars labeled with orange shows the extent of gene expression. The detailed information for all chromosomes of both cell lines is available in Additional file 8. The methylation profile for most genomic loci is very similar in both cell lines. Thus, the bars frequently show symmetric patterns in the figure.

The correlation between the methylation state of DNA fragments and their CNV was investigated (see Additional file 11). As illustrated in the plot, CNV seems to be independent of the DNA methylation state. By statistical analysis, we did not find any overall correlation between these two features in our study (data not shown).

**Figure 4:**
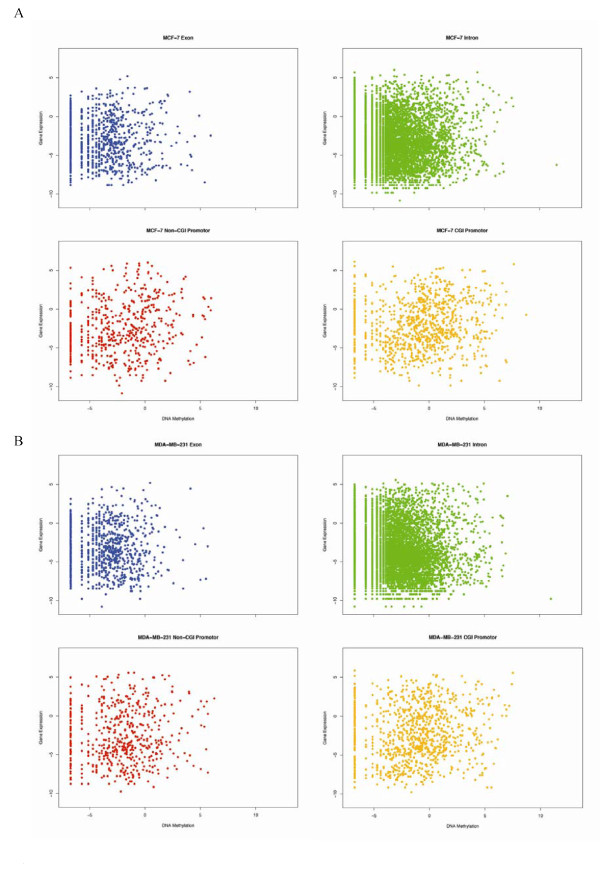
**Correlations between gene expression and DNA methylation**. Figure A and B present the plots of the relationships between the gene expression and the methylation of promoters with CGI, promoters without CGI, exons, and introns for MCF-7 and MDA-MB-231 cells, respectively. On the X-axis scale, the degree of methylation for each spot is calculated as the number of tags for a given site divided by the average number of tags for all *MluI *sites. Similarly, the degree of gene expression on the Y-axis scale is calculated as the expression value for a given gene divided by the average expression value for all genes. Both the methylation and expression values are log-transformed.

Furthermore, we explored the relationships between the methylation state of promoters, first exons and introns and the corresponding gene expression pattern. Significant correlations between the methylation state of promoters with CGIs and the corresponding gene expression were identified in MCF-7 (*R*^2 ^= 0.1461032, *P*-value: 4.845e-06) as well as in MDA-MB-231 (*R*^2 ^= 0.1238432, *P*-value: 0.0001093), respectively. Additionally, we found a similar significant correlation between the methylation state of the promoters lacking CGIs and gene expression in MCF-7 (*R*^2 ^= 0.14512, *P*-value: 0.0007437). Interestingly, we did not find such a correlation in MDA-MB-231 cells (*R*^2 ^= 0.06501603, *P*-value: 0.1324). No significant correlation was found between gene expression and the methylation of the first exon and intron for either of the cell lines in our study (data not shown). The plots of gene expressions and methylation profiles of the different sequence categories (CGIs promoters, first exons and introns) are presented in Fig 4.

The original data including array CGH, clone sequencing and bisulfate PCR sequencing are deposited in our public website "Snap": . Solexa sequencing results for both cell lines can be provided on request.

## Discussion

### Advantages of MMSDK

The combination of bisulfite modification and sequencing is the method of choice for methylation mapping in the Epigenome Project [27,28]. However, the high cost, labor-requirements and limitations of DNA fragment length (usually less than one kilo-base) hamper the application of this method in most laboratories. MSDK, as another sequence-based method, can exactly identify differently methylated sites genome-wide according to the selected enzyme(s), without the requirements of complex primer design, variable bisulfite modification and large-scale sequencing [12,13]. Compared to microarray-based assays, MSDK can analyze repeat sequences that exceed the dynamic detection range or show cross-hybridization in array analysis [29]. Furthermore, sequence-based methods seem to be more sensitive than microarray-based methods, because the former can directly detect unmethylated sequences at the single-base level, whereas the latter identify methylation state through capture of fluorescence signals by hybridization. However, the original MSDK is relatively low-throughput, labor-intensive and expensive due to the limitation of the sequencing approach [13]. To address these issues, we developed MMSDK, employing the Solexa 1G Analyzer. This new sequencer can produce 1 Gigabase of sequence per run (one run consists of 8 lanes, and each lane is expected to yield up to 5 million tags (reads)). Moreover, it allows 48-hour unattended operation. Therefore, this approach dramatically increases throughput for a relatively low cost. We anticipate that MMSDK will pave the way for comprehensive use of sequencing in DNA methylation studies.

The sequencing results validated the affinity between biotinylated linker and streptavidin conjugated beads, showing that the collection of methylated DNA fragments is specific and reliable. By the validations with a qPCR based and bisulfite treated PCR and clone sequencing methods, the reliability of the MMSDK method could be largely confirmed, although some variations were observed. The reasons for these quantitative variations between the outcomes from MMSDK and qPCR and bisulfite clone sequencing based methods might be due to imperfect cleavage by *MluI *and/or a bias derived from the PCR amplification used in MMSDK and a bias from qPCR itself (for qPCR), as well as a random selection of clones (for bisulfite clone sequencing). Our present study demonstrated MMSDK using *MluI *enzyme as an example. In practice, this method can easily be expanded to achieve a more comprehensive map of quantitative methylation profiles by using a variety of methylation sensitive restriction enzymes in individual or combinatory formats.

### Overview of the distribution of unmethylated sites for MCF-7 and MDA-MB-231 cells

Our present study showed a general similarity of the methylation profiles for promoters (with and without CGIs), exons, introns, and CGIs (excluding promoters) in the MCF-7 and MDA-MB-231 cell lines. However differences were present in introns (note that only 52 sites were analyzed in this study) indicated a significantly different distribution of unmethylated sites between the two cell lines. The small number of *MluI *recognition sites in this analysis could be a source of bias and the tendency to lesser methylation of introns in MDA-MB-231 cells needs further examination. The overall similarity of the distribution of unmethylated sites between two cell lines in our study is consistent with the results from Shann et al. [4]. They compared the distribution of unmethylated sites with respect to the positions of promoter, 1^st ^exon, 1^st ^intron, inner region of gene, and regions containing no transcribed sequence in MCF-7, MDA-MB-231, and normal human tissue genomes. In their study, a highly similar methylation pattern in MCF-7 and MDA-MB-231 was identified. However, this pattern was significantly different from those patterns identified in normal breast tissues [4].

### Impact of DNA methylation on genomic stability and gene expression

Regional DNA hypermethylation and global hypomethylation seem to be involved in different stages of breast cancer development [30,31]. Functional outcomes of hypomethylation include the transcriptional up-regulation of proto-oncogenes, and increased genomic instability [32]. A correlation between hypomethylation status of repeat sequences and genomic instability was reported in previous studies [33]. Only a small proportion of the *MluI *sites in the human genome are located in repeat sequences, but we collected many tags from these repeat sequences, showing that they are present in an unmethylated state. Considering the obvious genomic instability in both cell lines, our results support a correlation between hypomethylation in repeat elements and genome instability. Gilbert et al reported that LINE-1 retrotransposition could result in significant deletions of genomic sequence [34]. Hypomethylation of LINE-1 in tumor cells in comparison to normal tissues was previously described [33,35,36], and a decrease in the level of methylation of LINE-1 was suggested to activate transcription and increase retrotransposition events [33]. Notably, although a considerable number of tags were found in both the MCF-7 and MDA-MB-231 cell lines, there was a significant higher LINE-1 demethylation level in MDA-MB-231 compared to MCF-7. A higher percentage of deletions was observed among the observations in MDA-MB-231 cells (33.78%) compared to MCF-7 cells (19.58%). These observations could lead support to the idea that hypomethylation of LINE-1 has an impact on global genome deletion processes in tumors.

Common fragile sites are sites of frequent breakages and rearrangements in tumor cells [37]. In our study, 20% and 22% genome breakpoints were found to be located in the neighborhood of common fragile sites in MCF-7 and MDA-MB-231, respectively. It is hypothesized that breaks at fragile sites may serve as signatures of stalled or delayed replication in tumor cells, aggravated by deficiencies in the S-phase and G2/M checkpoints or associated repair genes during tumorigenesis [37,38].

Most cytosines within CpG dinucleotides are methylated in the human genome, but some remain unmethlyated in CpG islands [39]. Regional hypermethylation in tumorigenesis typically occurs in CGIs located in gene promoter regions [32]. Results of hypermethylation include suppression of tumor suppressor genes and chromatin condensation [32]. In the genome-wide analyses presented here, we indeed find that gene expression shows a stronger correlation with the methylation state of the corresponding CGIs in promoters than those in other components of the genes. Rivenbark and his colleagues reported that putative genes whose expression is under methylation-dependent regulation include genes lacking typical CGIs, based on their study on the MCF-7 cell line using a microarray-based methylation method [40]. Besides a significant correlation between gene expression and the methylation of the promoters with CGIs, we also find a significant correlation between gene expression and the methylation of the promoters without CGIs in this cell line, supporting Rivenbark's observations [40]. However, a similar correlation did not appear in the analysis in MDA-MB-231 cells suggesting cell type specific differences. We also noticed that the correlation of gene expression and methylation state of CGIs in promoter regions is not very high in both cell lines, although it is significant and stronger than the other correlations. A reasonable explanation is that not all genes of the genome are subject to DNA methylation-dependent regulation, and that gene regulation mainly depends on multiple regulation mechanisms.

### Luminal-like and mesenchymal-like cellular subtypes

Breast cancer classification has recently evolved with the definition of molecular subtypes with different prognosis based on large-scale gene expression profiling [7,41] and protein expression profiling [42,43]. Breast cancer cell lines have also been classified in molecular subtypes according to their expression profiles [7,44]. Recently, based on the analysis of the gene expression profile in 31 breast cell lines, "luminal" and "mesenchymal" subtype features were revealed [6]. MCF-7 and MDA-MB-231 cell lines both originate from pleural effusion metastatic cells from ductal invasive breast carcinomas [10]. However, MCF-7 expresses large numbers of markers typical of the luminal epithelium, presenting a "luminal-like" phenotype, whereas MDA-MB-231 is presenting a "mesenchymal-like" phenotype [10]. MCF-7 and MDA-MB-231 are among the most commonly used breast cancer cell lines in laboratories. As a result, MCF-7 and MDA-MB-231 had 5774 and 1157 citations, respectively, with some of the highest citation frequencies compared with other breast cancer cell lines in the past decade of cancer research [9]. Therefore, MCF-7 and MDA-MB-231 cells are regarded as good representatives to use for comprehensive analyses of the similarity and difference in biological nature between luminal-like and mesenchymal-like cancer subtypes. Accordingly, the DNA methylation patterns of these two cell lines revealed in this study might be useful for other researchers.

Charafe-Jauffret et al, identified 629 transcripts that are expressed preferentially in the group of luminal-like breast cancer cell lines, and 680 transcripts preferentially expressed in the group of mesenchymal-like cell lines [6]. In our expression study, 328 transcripts were identified with significantly higher expression in MCF-7 cells and overlapping with the luminal subtype gene list from Charafe-Jauffret's study [6]. 387 transcripts were identified with significantly higher expression in MDA-MB-231 cells which were also identified in the mesenchymal-like subtype gene list in Charafe-Jauffret's study [6]. Accordingly, to some extent, our expression results correlate the findings of the luminal and mesenchymal subtype makers in Charafe-Jauffret's study. Thus we investigated whether the DNA methylation status of CGIs correlate with gene expression for genes involved in determining the luminal and mesenchymal profiles of the two cell lines. We analyzed the DNA methylation states only for genes identified both in our expression analysis and in the analysis by Charafe-Jauffret. From these 715 transcripts we could deduce the CGI methylation status for 42 genes and derive candidate genes whose CGI hypomethylation level is consistent with the expression level by comparison of the two cell lines. These candidate genes includes *ARSJ, C10orf56, FLRT2, IGF2BP3 *and *PDGFC *representing luminal cell expression, and *MCF2L, ARRDC4, PREX1, TSGA2, CTNND2, TPD52L1, TRPS1 *and *RBM35A *representing mesenchymal cell expression. If CGI methylation indeed is involved in the transcriptional regulation of these candidate genes will be an important issue for our future research. Thus, our study indicates the possibility to use MMSDK not only to determine methylation profiles between different genome samples but also to correlate DNA methylation profiles with gene expression and gene CNV data. From the basis of this study the future employment of methylation-sensitive enzyme(s) that have more recognition sites in the human genome could provide more detailed information of DNA methylation profiles of individual genes.

### Conclusion

We have developed an improved approach, MMSDK, to explore DNA methylation patterns genome wide and applied this approach to the analysis of two representatives of luminal-like and mesenchymal-like cell lines. The data derived from MMSDK allows epigenomic profiling that can be combined with genomic CNV and gene expression data produced by microarray-based high-throughput approaches to achieve a comprehensive "Omics"-level analysis. We also addressed the impact of DNA methylation on gene expression for the two cancer cell lines and identified potential candidate genes whose expression might be regulated by methylation of their CGIs.

## Abbreviations

MSDK: methylation-specific digital karyotyping; MMSDK: modified methylation-specific digital karyotyping; CNV: copy number variation; SAGE: serial analysis of gene expression; SBS: Sequencing-By-Synthesis; aCGH: array comparative genomic hybridization; bp: base-pair; CGIs: CpG islands; MAQ: mapping and assembly with qualities; FDR: False discovery rate; FWER: familywise error rate; LINE: long interspersed nuclear element; TSSs: transcript start sites.

## Competing interests

The authors declare that they have no competing interests.

## Authors' contributions

JL performed the experiments of MMSDK, array CGH, gene expression microarray, analyzed the data, organized the work and drafted the manuscript. JL and FG performed qPCR and bisulfite clone sequencing. FG designed MMSDK, optimized the protocol and joined the discussion. NL performed bioinformatic and statistical analyses, joined the discussion and data analysis and helped in modification of manuscript. SL, GY and KW assisted in bioinformatic analysis and helped in figures. GT helped in MMSDK design and Solexa sequencing. SJ helped in clone sequencing and joined the discussion. XZ helped in Solexa sequencing. HY joined in discussion. ALN designed qPCR assay, joined the discussion and data analysis, helped in modification of the manuscript. LB organized the collaboration, joined the discussion and helped in modification of manuscript. All authors read and approved the final manuscript.

## Supplementary Material

Additional File 1 **The sequences of linkers and primers**. This file provides all sequences of linkers and primers used in our MMSDK approach. Click here for file

Additional File 2 **The strategy of the validation of methylation status with qPCR**. Genomic DNA from two cell lines was equally divided into two parts: one portion was digested with *MluI *; the other portion served as an undigested control. The digested and undigested DNA samples were amplified by qPCR using locus-specific primers that flank a given *MluI *restriction site in the human genome. The methylation state of a given genomic locus is determined by the failure of PCR amplification of the *MluI *digested fragments. By contrast, the fragments containing methylated *MluI *sites are protected from the digestion and, thus, serve as templates for amplification. Click here for file

Additional File 3 **The information of genomic loci and primers for qPCR and bisulfite clone sequencing**. This file provides the information of genomic loci that are selected for the validation of MMSDK using qPCR and bisulfite clone sequencing, as well as the sequences of the primers of qPCR and bisulfite clone sequencing. Click here for file

Additional File 4 **The distribution of sequence tags**. Figure A presents the distribution of the length of DNA fragments digested with *MluI *in a digital enzyme cutting simulation. Figure B shows the length distribution of the tags (reads) obtained from the Solexa 1G Genome Analyzer and the mapping results for MCF-7 and MDA-MB-231 cells, respectively. Click here for file

Additional File 5 **Summary of methylation status, expression value, DNA copy number variation and the information of genomic locus for each *MluI *site in the genome of MCF-7 and MDA-MB-231 cells**. The file provides all the results of MMSDK, gene expression and array CGH. The annotations of the headers are available in the bottom of this file. Click here for file

Additional File 6 **The validation of the methylation states for given genomic loci by qPCR**. 12 genomic loci were selected for methylation status validation. The information for genomic loci on the X-axis is indicated in the format the order number of genomic locus/tag number in MCF-7/tag number in MDA-MB-231. The Y-axis shows the relative amount of DNA in each sample. The presented data are the result of triplicate PCR amplifications. Blue indicates genomic DNA of MCF-7 digested with *MluI*; red indicates MCF-7 without *MluI *enzyme; green indicates MDA-MB-231 genomic DNA digested with *MluI*; and purple MDA-MB-231 without *MluI*. The height of each column represents the amount of initial amount of template estimated by qPCR. The last template is the control containing a *HindIII *cutting site (AAGCTT) but no *MscI *cutting site (TGGCCA). Click here for file

Additional File 7 **The validation of the methylation states for given genomic loci by bisulfite treated PCR and clone sequencing**. Five randomly selected representative *MluI *genomic loci were preformed bisulfite treated PCR and clone sequencing. Seven clones were sequenced for each genomic locus in the both cell lines. Each line shows the result of one clone sequencing, in which each circle presents a CpG dinucleotide. The solid circles present methylated and the empty unmethylated. The red rectangles indicate *MluI *sites, and the methylation statuses of neighbor CpGs are also shown in the figure. The numbers of tags collected by MMSDK for each genomic locus in each cell line are shown in the figure. Click here for file

Additional File 8 **The information of methylation, CNV and expression on all chromosomes**. This file contains 22 figures to present the results of methylation, DNA copy number variation and gene expression for 22 (chr1-chrX) individual chromosomes for both cell lines. Click here for file

Additional File 9 **The genes show preferential expression in MCF-7**. This file display all genes that express preferentially in MCF-7. Click here for file

Additional File 10 **The genes show preferential expression in MDA-MB-231**. This file display all genes that express preferentially in MDA-MB-231. Click here for file

Additional File 11 **Correlations between DNA methylation and DNA copy number variation**. The plot presents the relationships between methylation and DNA copy number variation for MCF-7 cells (red) and MDA-MB-231 cells (blue). On the X-axis scale, the value of methylation for each spot (*MluI *site) is calculated by dividing the number of tags for a given site in a cell line by the average number of tags of all *MluI *sites in the cell line. The value of DNA copy number variation (CNV) on the Y-axis scale is calculated as the ratio for a given DNA dosage within a cell line to that in normal reference DNA. Both the methylation and CNV values are log-transformed. Click here for file
