# Supplementary material for: An improved method for genome wide DNA methylation profiling correlated to transcription and genomic instability in two breast cancer cell lines
Source: BMC Genomics. 2009 May 13;10:223. doi: 10.1186/1471-2164-10-223 (PMC2696471; doi:10.1186/1471-2164-10-223)
Supplement: Additional File 1 — The sequences of linkers and primers. This file provides all sequences of linkers and primers used in our MMSDK approach. [file 1471-2164-10-223-S1.doc]

**The sequences of linkers and PCR primers and the recognition sites of *MluI*, *NlaIII* and *MmeI* enzymes in MMSDK analysis**

*MluI* recognition site 5’-...A’ CGCG T…3’

3’-…T GCGC’ A…5’

Biotinylated linker:

5’-**Biotin**-TTTGCAGAGGTTCGTAATCGAGTTGGGTGG-3’

3’-CGTCTCCAAGCATTAGCTCAACCCACC GCGC-5’

*NlaIII* recognition site 5’…CATG’…3’

3’…’GTAC…5’

Linker N: 5’-ACAGGTTCAGAGTTCTACAG**TCCGAC**CATG

3‘-CAAGTCTCAAGATGTC**AGGCTG**

*MmeI* recognition site 5’…TCCRAC(N)_20_’…3’

3’…AGGYTG(N)_18_’…5' R=A or G; Y=C or T

P7 Linker: 5’-TCGTAT GCCGTCTT CTGC TTG-3’

3’-NNAGCATACGGCAGAAGACGAAC-5’

Final linkage product as template for the subsequent PCR

Linker N------tag 16 bp------Linker P7

5’-ACAGGTTCAGAGTTCTACAG**TCCGAC**CATG-NNNNNNNNNNNNNNNN- TCGTAT GCCGTCTT CTGC TTG-3’

3‘-CAAGTCTCAAGATGTC**AGGCTG**-GTACNNNNNNNNNNNNNN-NNAGCATACGGCAGAAGACGAAC-5’

PCR primers:

P5 long primer: 5’-AATGATACGGCGACCACCGACAGGTTCAGAGTTCTACAGTCCGA-3’

P7 primer: 5’-CAAGCAGAAGACGGCATACGA-3’

Final PCR product

5’-AATGATACGGCGACCACCGACAGGTTCAGAGTTCTACAGTCCGA-C CATG-NNNNNNNNNNNNNNNN-TCGTAT GCCGTCTT CTGC TTG-3’

3’-TTACTAT GCCGCTGGTGGC TGTCCAAGTCTCAAGATGTC**AGGCTG**-GTACNNNNNNNNNNNNNN-NNAGCATACGGCAGAAGACGAAC-5’

*MluI* recognition site 5’-...A’ CGCG T…3’

3’-…T GCGC’ A…5’

Biotinylated linker:

5’-**Biotin**-TTTGCAGAGGTTCGTAATCGAGTTGGGTGG-3’

3’-CGTCTCCAAGCATTAGCTCAACCCACC GCGC-5’

*Age I* recognition site 5’-...A’ CCGG T…3’

3’-…T GGCC’ A…5’

Biotinylated linker:

5’-**Biotin**-TTTGCAGAGGTTCGTAATCGAGTTGGGTGG-3’

3’-CGTCTCCAAGCATTAGCTCAACCCACC GGCC-5’

*AscI*  recognition site 5’-...GG’ CGCG CC…3’

3’-…CC GCGC’ GG…5’

Biotinylated linker:

5’-**Biotin**-TTTGCAGAGGTTCGTAATCGAGTTGGGTGG-3’

3’-CGTCTCCAAGCATTAGCTCAACCCACC GCGC-5’

*BsiWI*  recognition site 5’-...C’ GTAC G…3’

3’-…G CATG’ C…5’

Biotinylated linker:

5’-**Biotin**-TTTGCAGAGGTTCGTAATCGAGTTGGGTGG-3’

3’-CGTCTCCAAGCATTAGCTCAACCCACC CATG -5’

*BsrFI*  recognition site 5’-...R’ CCGG Y…3’

3’-…Y GGCC’ R…5’

Biotinylated linker:

5’-**Biotin**-TTTGCAGAGGTTCGTAATCGAGTTGGGTGG-3’

3’-CGTCTCCAAGCATTAGCTCAACCCACC GGCC-5’

*BssHII* recognition site 5’-...G’ CGCG C…3’

3’-…C GCGC’ G…5’

Biotinylated linker:

5’-**Biotin**-TTTGCAGAGGTTCGTAATCGAGTTGGGTGG-3’

3’-CGTCTCCAAGCATTAGCTCAACCCACC GCGC-5’

*EagI* recognition site 5’-...C’ GGCC G…3’

3’-…G CCGG’ C…5’

Biotinylated linker:

5’-**Biotin**-TTTGCAGAGGTTCGTAATCGAGTTGGGTGG-3’

3’-CGTCTCCAAGCATTAGCTCAACCCACC CCGG-5’

*HpaII*  recognition site 5’-...C’ CG G…3’

3’-…G GC’ C…5’

Biotinylated linker:

5’-**Biotin**-TTTGCAGAGGTTCGTAATCGAGTTGGGTGG-3’

3’-CGTCTCCAAGCATTAGCTCAACCCACC GC-5’

*KasI*  recognition site 5’-...G’ GCGC C…3’

3’-…C CGCG’ G…5’

Biotinylated linker:

5’-**Biotin**-TTTGCAGAGGTTCGTAATCGAGTTGGGTGG-3’

3’-CGTCTCCAAGCATTAGCTCAACCCACC CGCG-5’

*NarI*  recognition site 5’-...GG’ CG CC…3’

3’-…CC GC’ GG…5’

Biotinylated linker:

5’-**Biotin**-TTTGCAGAGGTTCGTAATCGAGTTGGGTGG-3’

3’-CGTCTCCAAGCATTAGCTCAACCCACC GC-5’

*NgoMIV*  recognition site 5’-...G’ CCGG C…3’

3’-…C GGCC’ G…5’

Biotinylated linker:

5’-**Biotin**-TTTGCAGAGGTTCGTAATCGAGTTGGGTGG-3’

3’-CGTCTCCAAGCATTAGCTCAACCCACC GGCC-5’

*NotI* recognition site 5’-...GC’ GGCC GC…3’

3’-…CG CCGG’ CG…5’

Biotinylated linker:

5’-**Biotin**-TTTGCAGAGGTTCGTAATCGAGTTGGGTGG-3’

3’-CGTCTCCAAGCATTAGCTCAACCCACC CCGG-5’

*PaeR7I*  recognition site 5’-...C’ TCGA G…3’

3’-…G AGCT’ C…5’

Biotinylated linker:

5’-**Biotin**-TTTGCAGAGGTTCGTAATCGAGTTGGGTGG-3’

3’-CGTCTCCAAGCATTAGCTCAACCCACC AGCT-5’

*SacII*  recognition site 5’-...CCGC’ GG…3’

3’-…GG’CG CC…5’

Biotinylated linker:

5’-**Biotin**-TTTGCAGAGGTTCGTAATCGAGTTGGGTGG-3’

3’-CGTCTCCAAGCATTAGCTCAACCCACC CG-5’

*SaII*  recognition site 5’-...G’ TCGA C…3’

3’-…C AGCT’ G…5’

Biotinylated linker:

5’-**Biotin**-TTTGCAGAGGTTCGTAATCGAGTTGGGTGG-3’

3’-CGTCTCCAAGCATTAGCTCAACCCACC AGCT-5’

*SgrAI* recognition site 5’-...CR’ CCGG YG…3’

3’-…GY GGCC’ RC…5’

Biotinylated linker:

5’-**Biotin**-TTTGCAGAGGTTCGTAATCGAGTTGGGTGG-3’

3’-CGTCTCCAAGCATTAGCTCAACCCACC GGCC-5’

*TspMI*  recognition site 5’-...C’ CCGG G…3’

3’-…G GGCC’ C…5’

Biotinylated linker:

5’-**Biotin**-TTTGCAGAGGTTCGTAATCGAGTTGGGTGG-3’

3’-CGTCTCCAAGCATTAGCTCAACCCACC GGCC-5’
