# Supplementary figures and images for: An improved method for genome wide DNA methylation profiling correlated to transcription and genomic instability in two breast cancer cell lines
Source: BMC Genomics. 2009 May 13;10:223. doi: 10.1186/1471-2164-10-223 (PMC2696471; doi:10.1186/1471-2164-10-223)

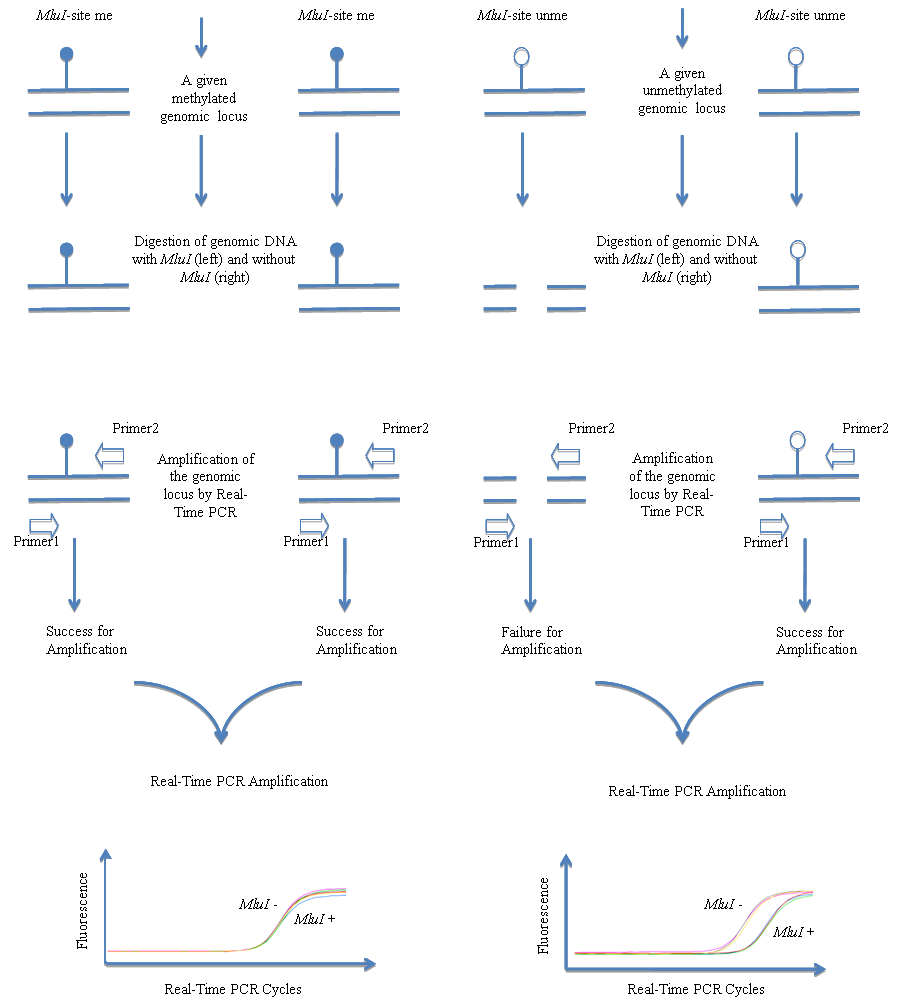

Supplement: Additional File 2 — The strategy of the validation of methylation status with qPCR. Genomic DNA from two cell lines was equally divided into two parts: one portion was digested with MluI ; the other portion served as an undigested control. The digested and undigested DNA samples were amplified by qPCR using locus-specific primers that flank a given MluI restriction site in the human genome. The methylation state of a given genomic locus is determined by the failure of PCR amplification of the MluI digested fragments. By contrast, the fragments containing methylated MluI sites are protected from the digestion and, thus, serve as templates for amplification. [file 1471-2164-10-223-S2.doc]

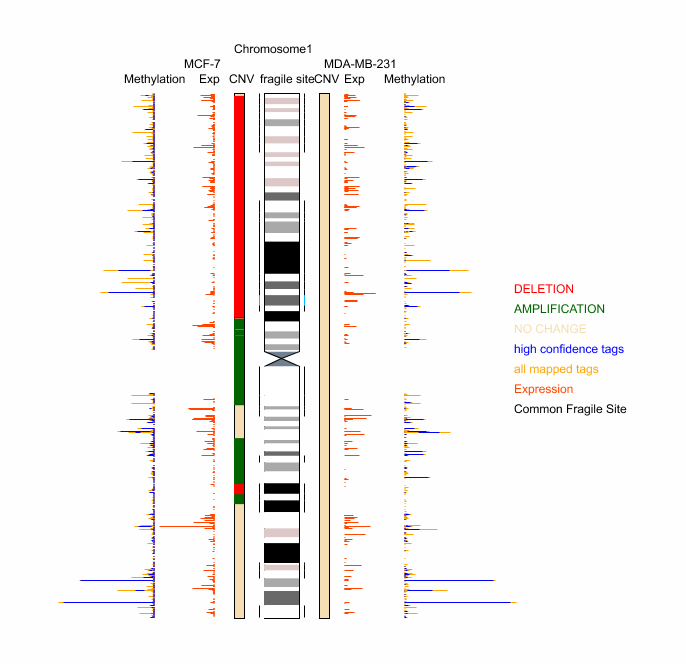


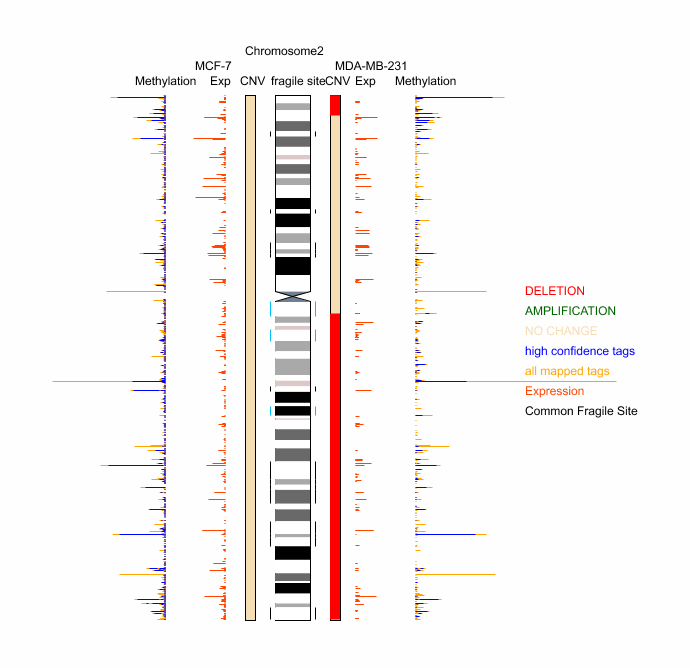


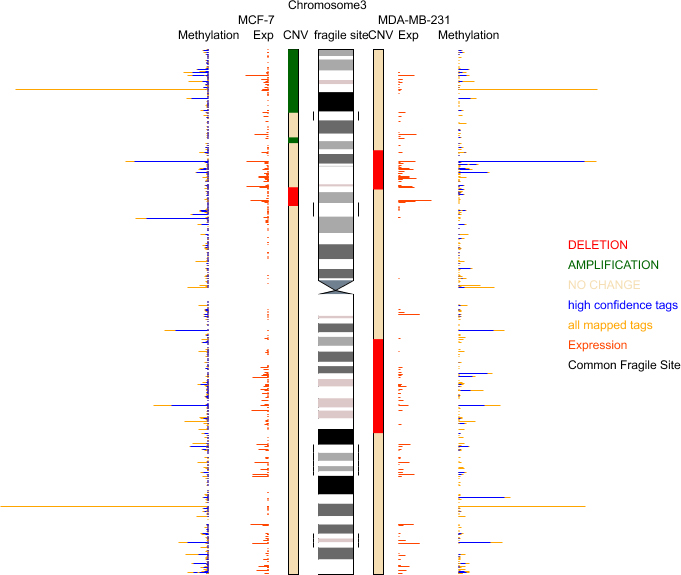


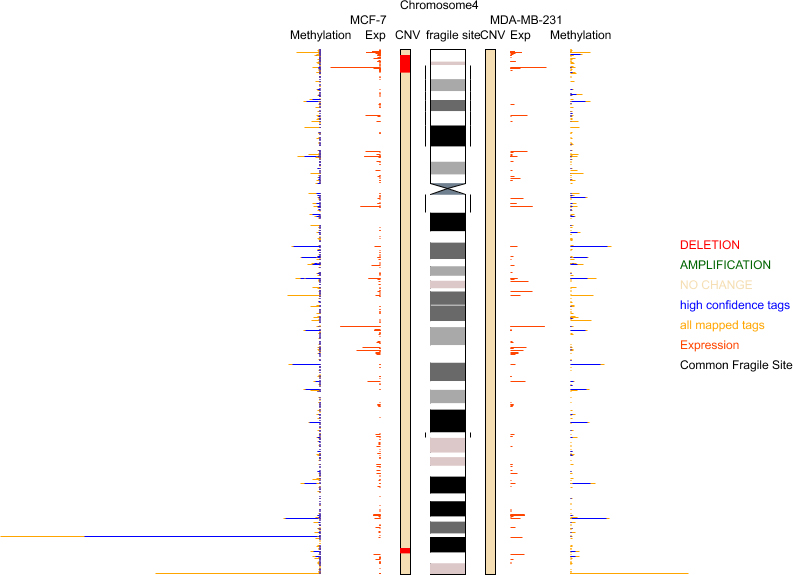


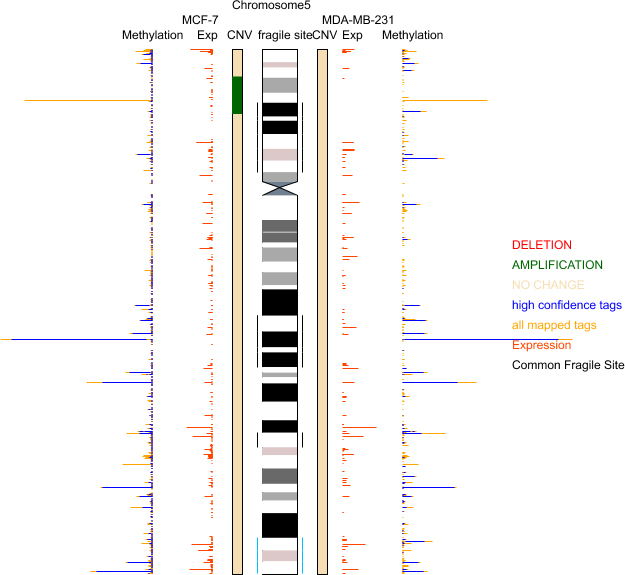


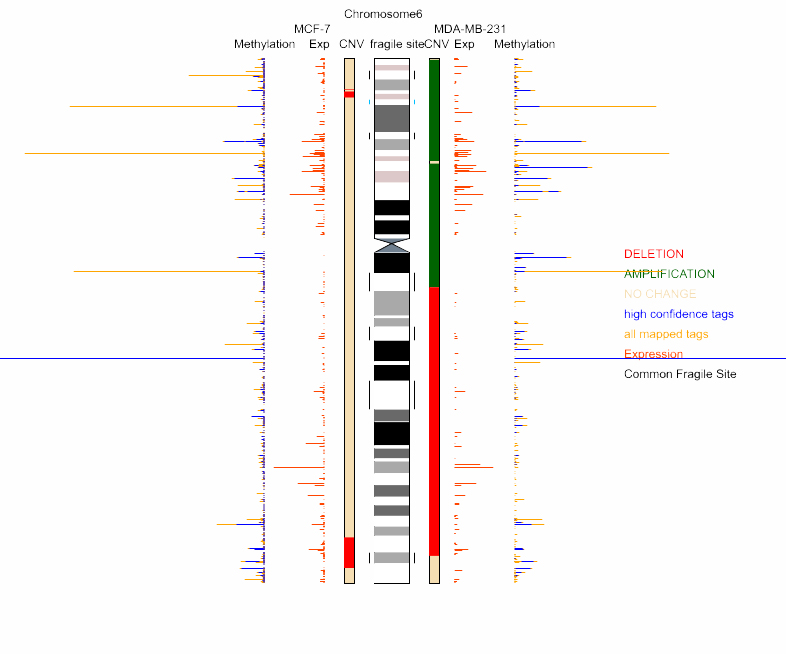


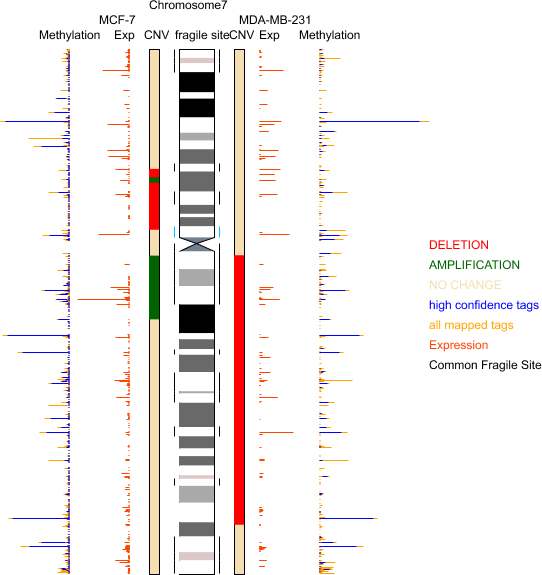


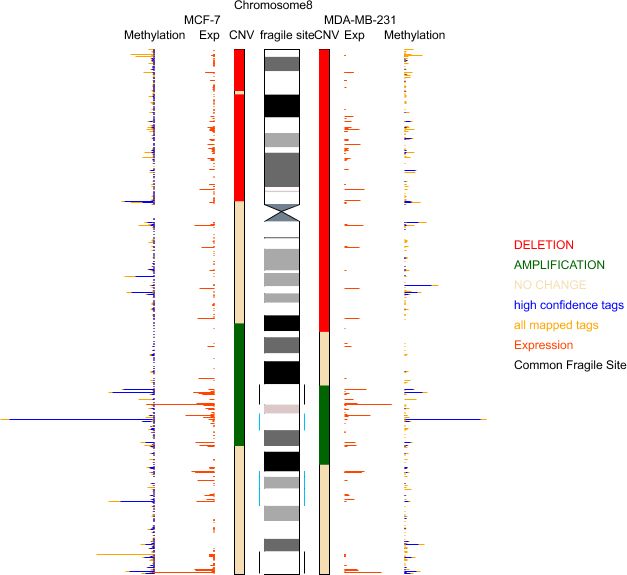


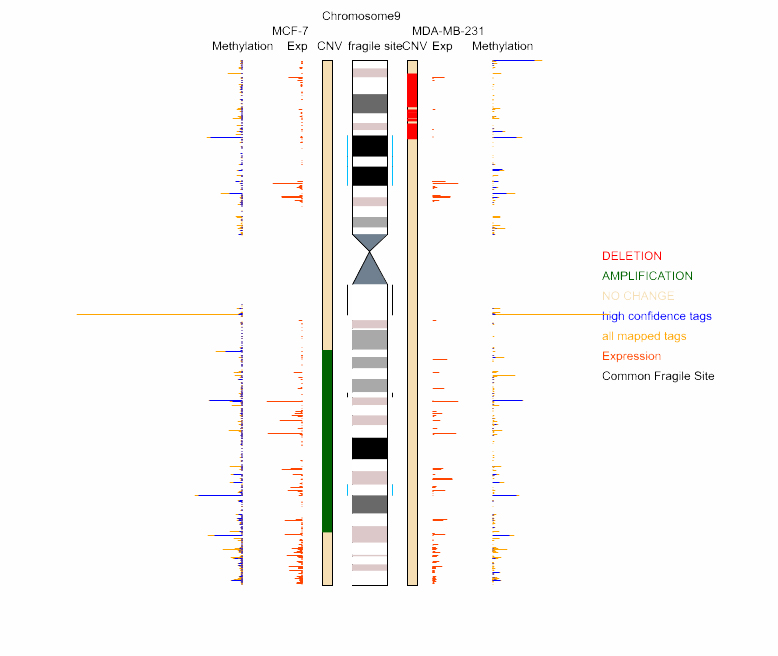


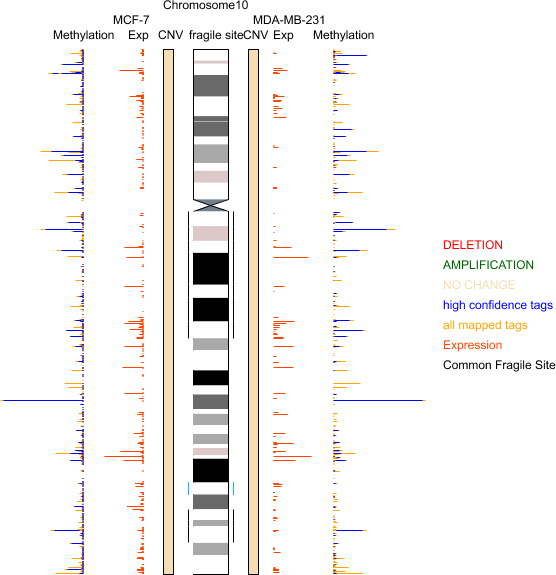


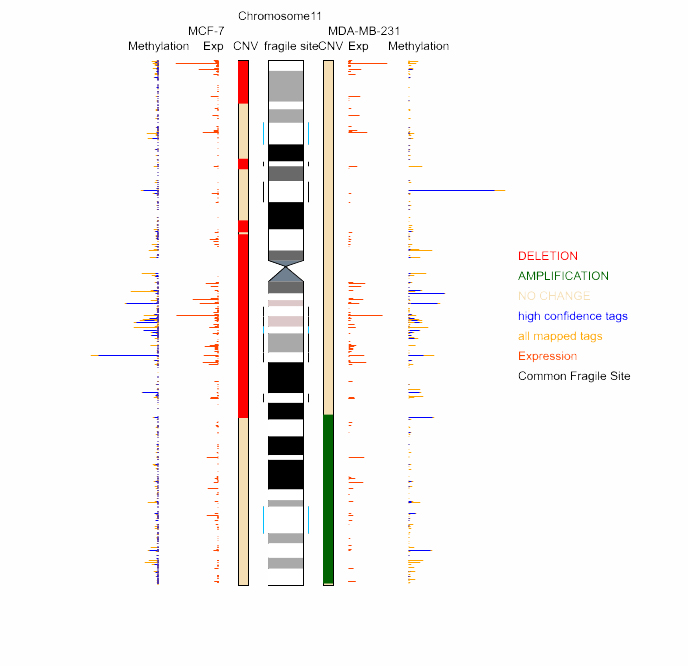


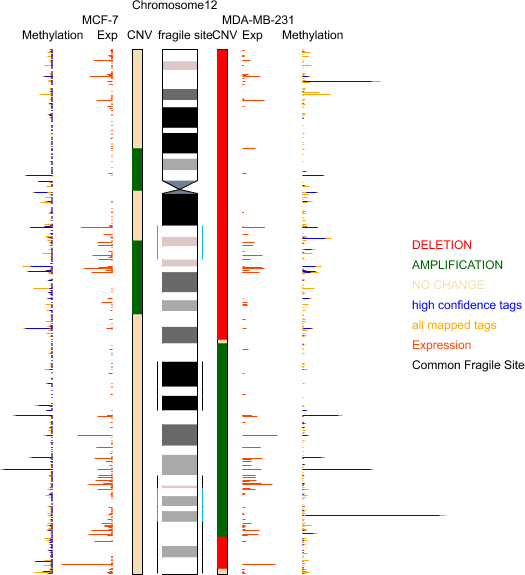


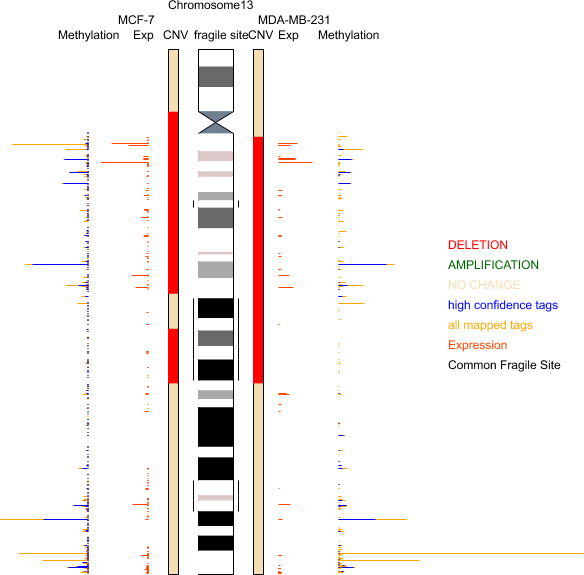


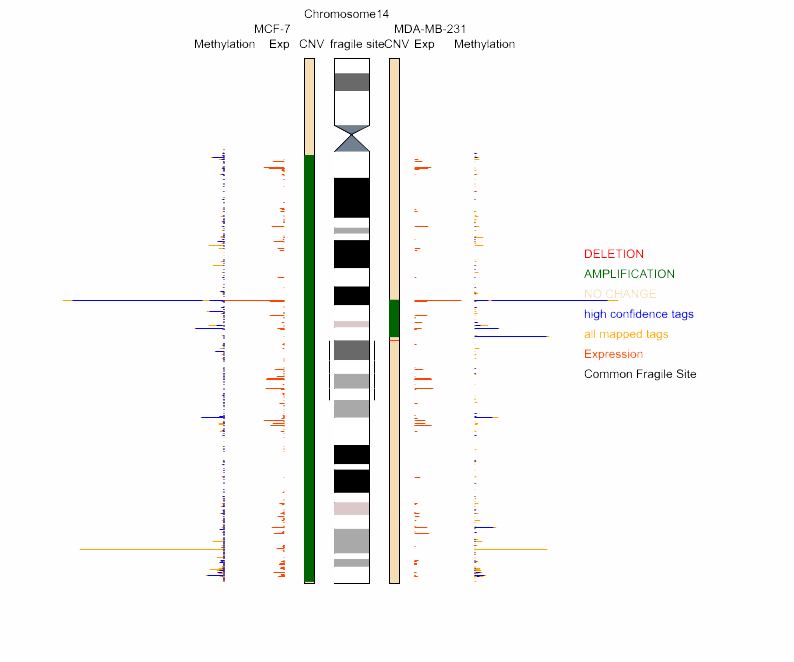


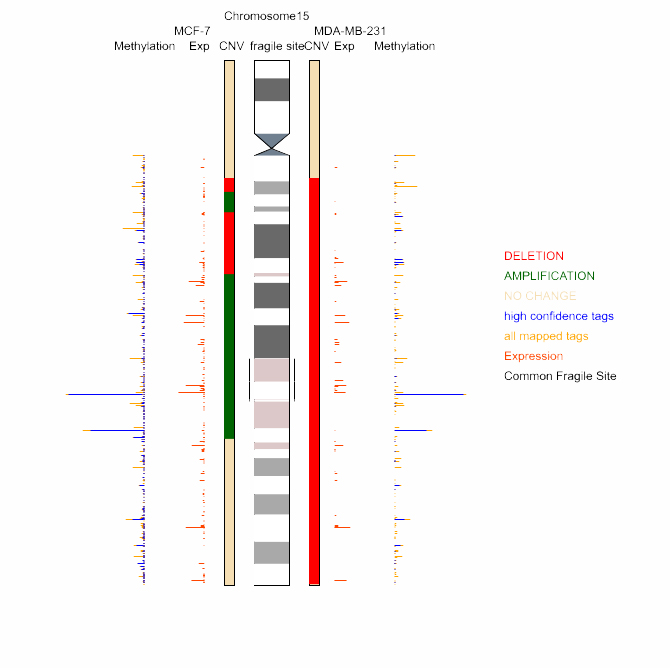


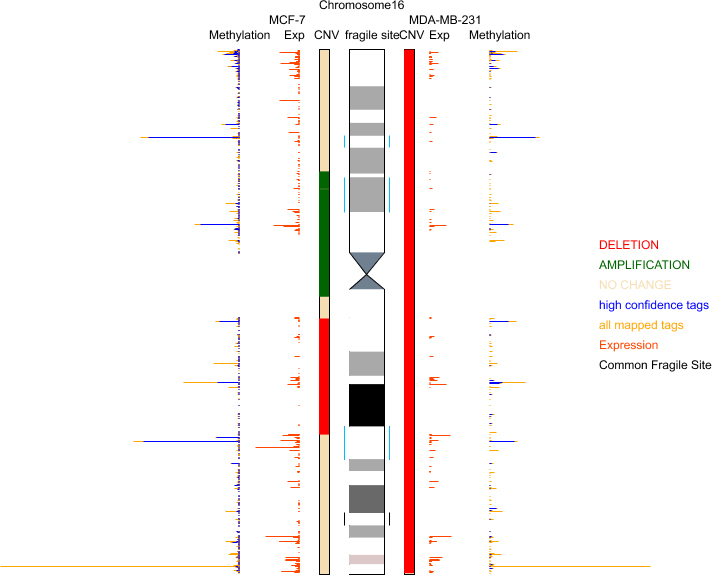


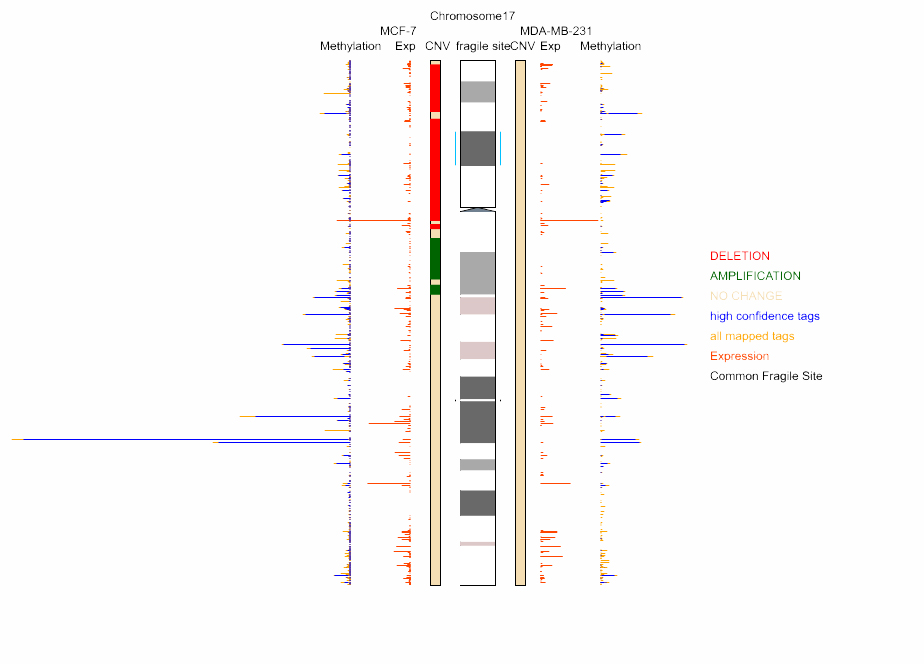


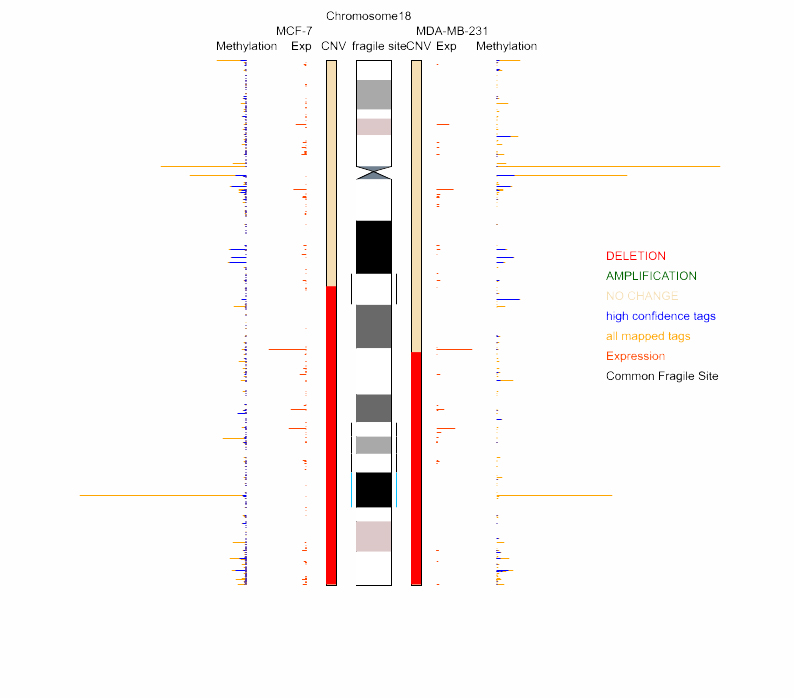


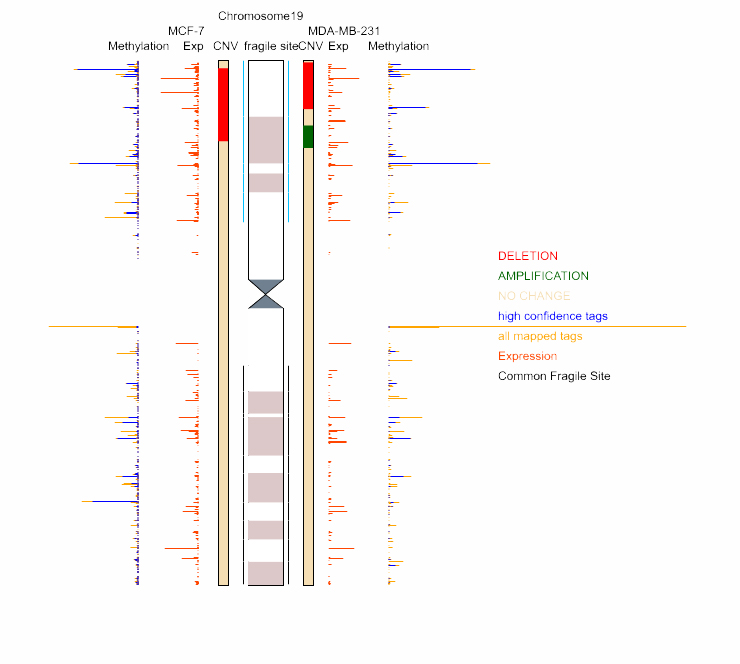


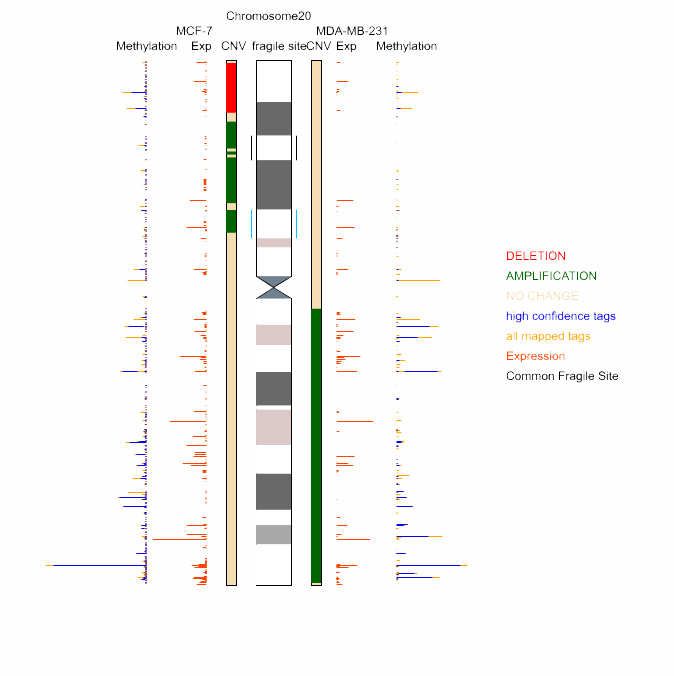


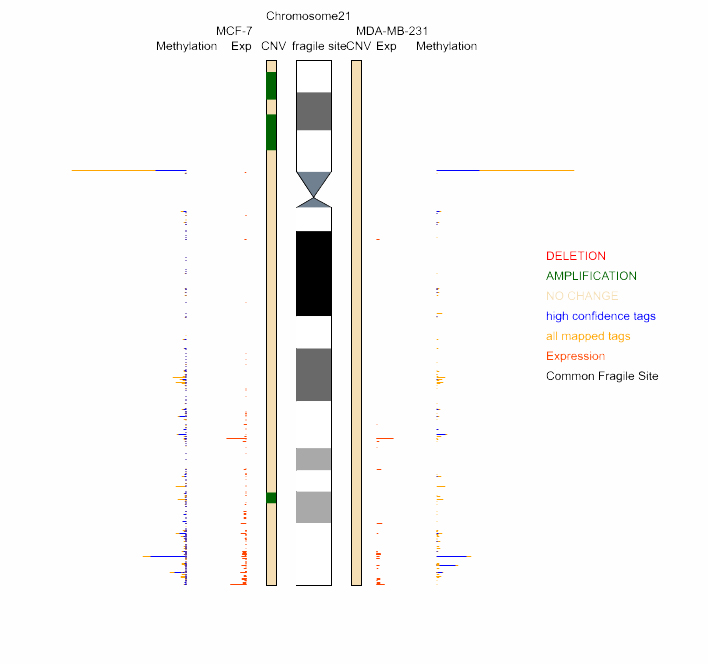


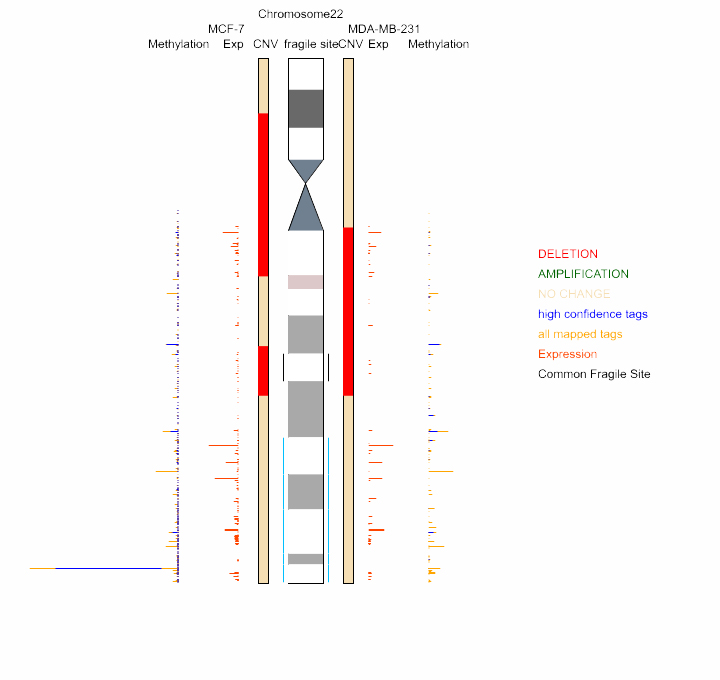


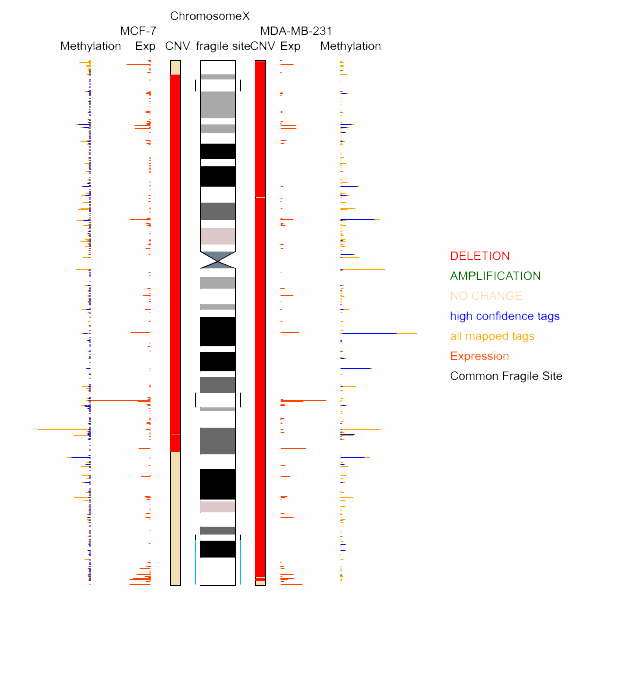

Supplement: Additional File 8 — The information of methylation, CNV and expression on all chromosomes. This file contains 22 figures to present the results of methylation, DNA copy number variation and gene expression for 22 (chr1-chrX) individual chromosomes for both cell lines. [file 1471-2164-10-223-S8.doc]

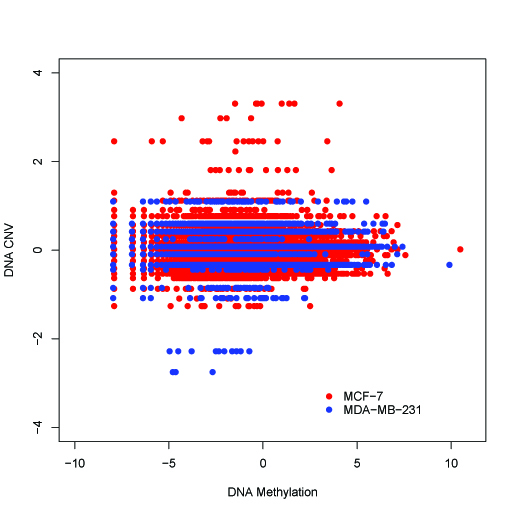

Supplement: Additional File 11 — Correlations between DNA methylation and DNA copy number variation. The plot presents the relationships between methylation and DNA copy number variation for MCF-7 cells (red) and MDA-MB-231 cells (blue). On the X-axis scale, the value of methylation for each spot (MluI site) is calculated by dividing the number of tags for a given site in a cell line by the average number of tags of all MluI sites in the cell line. The value of DNA copy number variation (CNV) on the Y-axis scale is calculated as the ratio for a given DNA dosage within a cell line to that in normal reference DNA. Both the methylation and CNV values are log-transformed. [file 1471-2164-10-223-S11.doc]
