# Supplementary material for: An improved method for genome wide DNA methylation profiling correlated to transcription and genomic instability in two breast cancer cell lines
Source: BMC Genomics. 2009 May 13;10:223. doi: 10.1186/1471-2164-10-223 (PMC2696471; doi:10.1186/1471-2164-10-223)
Supplement: Additional File 4 — The distribution of sequence tags. Figure A presents the distribution of the length of DNA fragments digested with MluI in a digital enzyme cutting simulation. Figure B shows the length distribution of the tags (reads) obtained from the Solexa 1G Genome Analyzer and the mapping results for MCF-7 and MDA-MB-231 cells, respectively. [file 1471-2164-10-223-S4.doc]

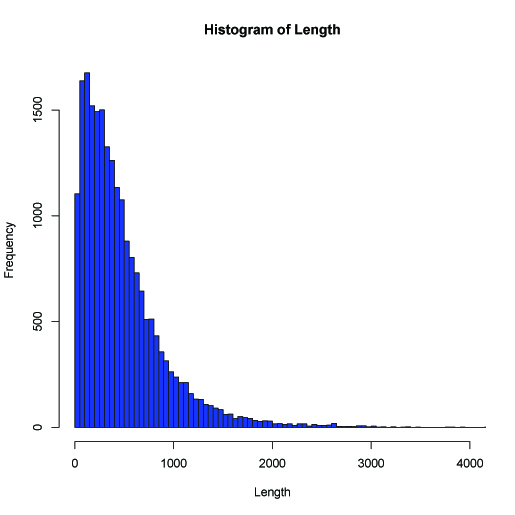
A

B

MCF-7: Total 16-17bp tags: 5432906. All mapped tags: 5127677, 94.38%; the mapped tags with high confidence: 2210449, 40.69%.

MDA-MB-231: Total 16-17bp tags: 5636928. All mapped tags: 5325000, 94.5%; the mapped tags with high confidence: 2185196, 38.8%.
