# Supplementary material for: An improved method for genome wide DNA methylation profiling correlated to transcription and genomic instability in two breast cancer cell lines
Source: BMC Genomics. 2009 May 13;10:223. doi: 10.1186/1471-2164-10-223 (PMC2696471; doi:10.1186/1471-2164-10-223)

No.1 chr9_38012335 MCF-7: 50, MDA-MB-231:12

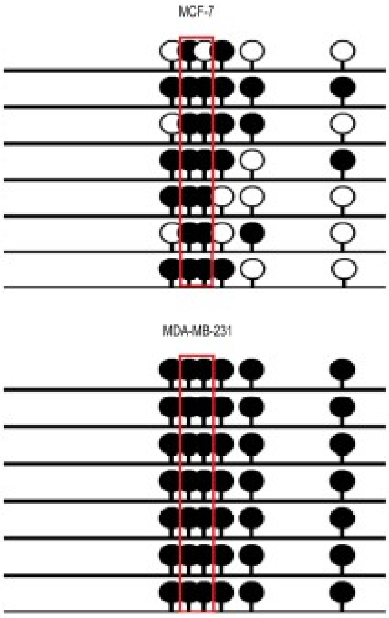

No.2a chr10_134815038 MCF-7: 10, MDA-MB-231:10

No.2b chr10_134815089 MCF-7: 10, MDA-MB-231:10

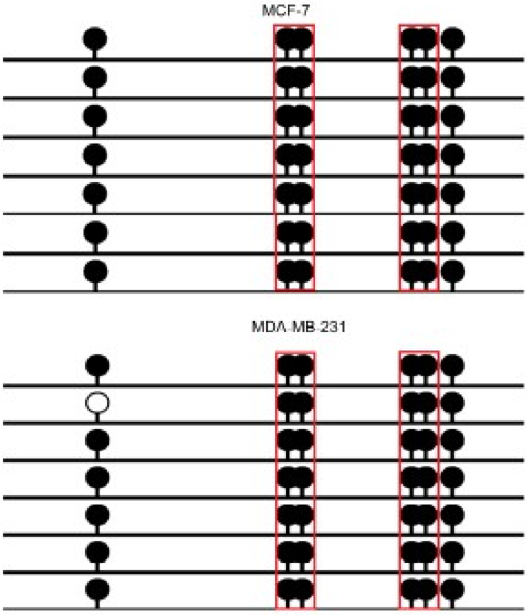

No.3 chr5_151131703 MCF-7:7082, MDA-MB-231:7546

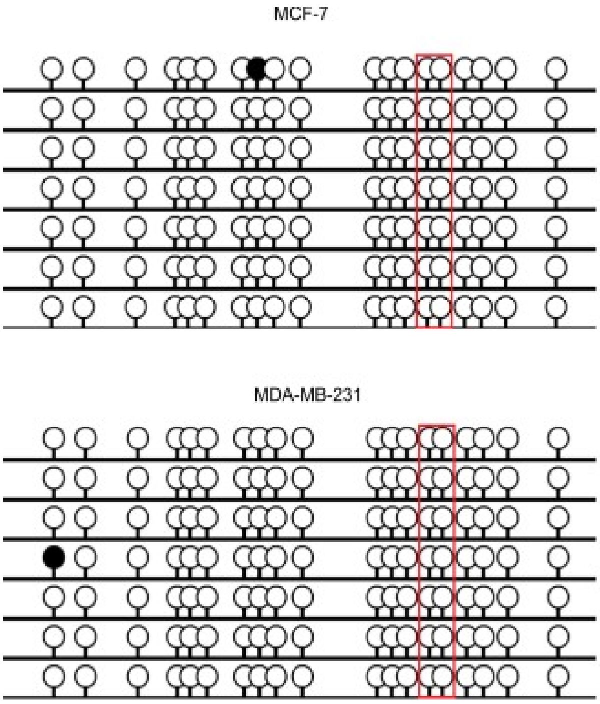

No.4 chr2_136592478 MCF-7:4514, MDA-MB-231:96

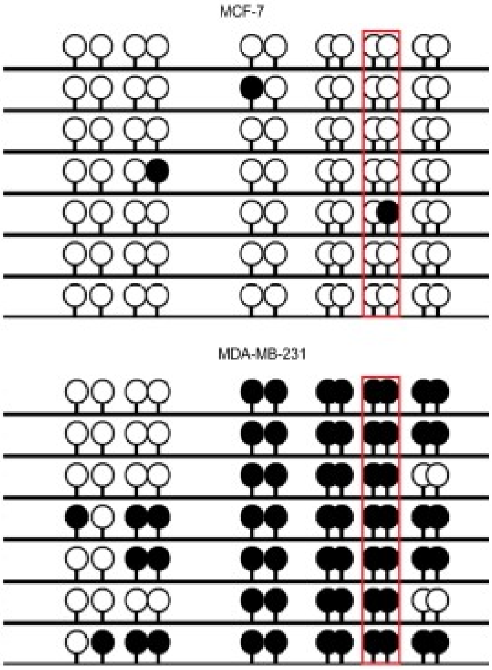

Supplement: Supplementary file 6 [file 1471-2164-10-223-S7.doc]
